# Enhanced Activity of P4503A4 and UGT1A10 Induced by Acridinone Derivatives C-1305 and C-1311 in MCF-7 and HCT116 Cancer Cells: Consequences for the Drugs’ Cytotoxicity, Metabolism and Cellular Response

**DOI:** 10.3390/ijms21113954

**Published:** 2020-05-31

**Authors:** Monika Pawłowska, Anna Kwaśniewska, Zofia Mazerska, Ewa Augustin

**Affiliations:** 1 Department of Pharmaceutical Technology and Biochemistry, Chemical Faculty, Gdańsk University of Technology, 80-233 Gdańsk, Poland; zofia.mazerska@pg.edu.pl (Z.M.); ewa.augustin@pg.edu.pl (E.A.); 2 Nencki Institute of Experimental Biology, Polish Academy of Sciences, 02-093 Warsaw, Poland; a.kwasniewska@nencki.edu.pl

**Keywords:** acridinone derivatives, anticancer drugs, P4503A4 isoenzyme, UGTs, enzymatic activity, drug metabolism, cytotoxicity, cellular response

## Abstract

Activity modulation of drug metabolism enzymes can change the biotransformation of chemotherapeutics and cellular responses induced by them. As a result, drug-drug interactions can be modified. Acridinone derivatives, represented here by C-1305 and C-1311, are potent anticancer drugs. Previous studies in non-cellular systems showed that they are mechanism-based inhibitors of cytochrome P4503A4 and undergo glucuronidation via UDP-glucuronosyltranspherase 1A10 isoenzyme (UGT1A10). Therefore, we investigated the potency of these compounds to modulate P4503A4 and UGT1A10 activity in breast MCF-7 and colon HCT116 cancer cells and their influence on cytotoxicity and cellular response in cells with different expression levels of studied isoenzymes. We show that C-1305 and C-1311 are inducers of not only P4503A4 but also UGT1A10 activity. MCF-7 and HCT116 cells with high P4503A4 activity are more sensitive to acridinone derivatives and undergo apoptosis/necrosis to a greater extent. UGT1A10 was demonstrated to be responsible for C-1305 and C-1311 glucuronidation in cancer cells and glucuronide products were excreted outside the cell very fast. Finally, we show that glucuronidation of C-1305 antitumor agent enhances its pro-apoptotic properties in HCT116 cells, while the cytotoxicity and cellular response induced by C-1311 did not change after drug glucuronidation in both cell lines.

## 1. Introduction

The metabolism of antitumor agents in patients has a significant impact on the final effects of their actions. Cellular responses induced by antitumor agents might be affected by the abundance and activity of drug metabolism enzymes. Cytochrome P450 is the main family of drug metabolism enzymes and provides most of the phase I biotransformations of endobiotics and xenobiotics. One P450 isoenzyme, P4503A4, takes part in the metabolism of more than 50% of all administered therapeutic agents [1], as well as the activation of many carcinogens [2]. Moreover, three main enzyme families are involved in the detoxification reaction of xenobiotics—UDP-glucuronosyltranspherases (UGTs), glutathione-*S*-transferases (GSTs) and the above mentioned cytochromes P450. However, the reaction of *O*-glucuronidation, catalyzed by UGTs, is considered to be crucial in that process [2].

**Figure 1:**
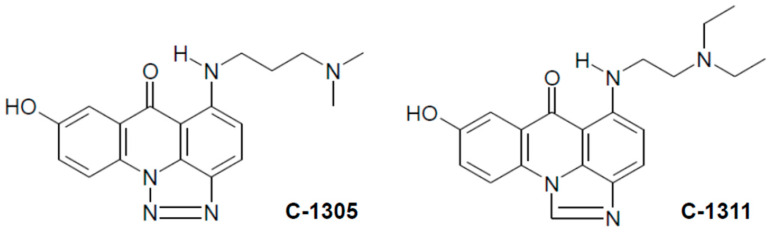
Chemical structures of studied compounds, triazoloacridinone (C-1305) and imidazoacridinone (C-1311).

Triazolo- and imidazoacridinone heterocyclic compounds, represented here by C-1305 and C-1311 compounds, respectively (Figure 1), exhibited antineoplastic properties in a wide spectrum of transplantable tumors [3]. C-1305 (5-dimethylaminopropylamino-8-hydroxytriazoloacridinone) and its analogs showed significant cytotoxic activity toward 64 human tumor cell lines in the National Cancer Institute screening system (Bethesda, MD, USA) and displayed high antitumor activity against several experimental tumors in mice, particularly leukemias and colon carcinomas [4,5]. C-1305 was selected for extended preclinical studies. The best known imidazoacridinone analog, C-1311 (5-diethylaminoethylamino-8-hydroxyimidazoacridinone, NSC-645809) [6], has shown activity against experimental models of murine and human colorectal cancers in vitro and in animals [7]. It was evaluated in phase I clinical trials in patients with advanced solid tumors [8,9] and was effective in a phase II clinical trial in women with metastatic breast cancer [10]. Other studies have shown that C-1311 in combination with paclitaxel was effective against human bladder cancer in an in vivo hollow fiber assay [11].

Studies on the biochemical mechanisms of C-1305 and C-1311 action have also been performed [12,13,14,15]. It has been shown that both compounds intercalated to DNA [16]; however, the crucial process for the observed antitumor activity was their inhibition of topoisomerase II activity [17,18] and interstrand covalent DNA cross-linking [19,20]. Additionally, C-1311 effectively decreased tumor angiogenesis by downregulating the hypoxia-inducible factor-1 α (HIF-1α)/vascular endothelial growth factor (VEGF) pathway [21] and blocked quinone oxidoreductase 2 (NQO2) [22]. Moreover, both compounds were shown to be potent and selective inhibitors of FMS-like tyrosine kinase 3 with internal tandem duplications mutations (FLT3-ITD) [23,24].

It was shown previously that although cytochromes P450 are not involved in C-1305 and C-1311 activation in non-cellular systems; both compounds were shown to be selective irreversible inhibitors of P4501A2 and P4503A4. This inhibition was shown to be mechanism-based for both compounds [25,26]. Each compound was also found to be metabolized with human recombinant flavin-containing monooxygenases 1 and 3 (FMO1, FMO3) [27,28]. Although the metabolism of both studied drugs did not depend on P4503A4 expression, the cellular response, including apoptosis, was stronger in P4503A4-overexpressing HepG2 and CHO cells following C-1305 [29,30] and C-1311 [31,32] treatment. It was also demonstrated that the studied compounds underwent UGT-mediated metabolism to 8-*O*-glucuronides with human liver and intestinal microsomes and particularly with recombinant extrahepatic UGT1A10 isoenzyme [33]. Moreover, studies performed in KB-3 cells transiently transfected with UGT1A10 demonstrated that glucuronidation of both compounds and UGT1A10 overexpression in KB-3 cells significantly increased the cytotoxicity of C-1305 but not C-1311. It should be underlined that the last conclusion contradicted the dogma, which indicates that glucuronidation results in deactivation of xenobiotics [34].

Considering drug metabolism, the level and activity of metabolic enzymes strongly influence the final patient response to drug therapy. Moreover, it should also be considered that the drug can affect enzyme activity. Therefore, it can also modulate the pharmacokinetics of other therapeutics in multidrug treatment. The correlation between different enzyme activity and patient response to therapy was shown for antitumor agents, for example, irinotecan [35], tamoxifen [36] and docetaxel [37]. What is more, the particular enzyme can influence the metabolism of endogenous compounds, which can also affect the effectiveness of the therapy, due to their influence on cell proliferation and different pathways of cell death. Such relationships were found for P4501B1 [38], P4503A4 [39], P4502J2 [40] and UGT2B15/17 [41].

Considering all of the above, the present study aimed to investigate, on the one hand, the impact of P4503A4 and UGT1A10 overexpression in breast MCF-7 and colon HCT116 cancer cells on C-1305 and C-1311 metabolism, and, on the other hand, their potency of modulating such enzyme activity following drug’s treatment. We chose breast MCF-7 and colon HCT116 cancer cells, as phase I clinical trials with C-1311 were performed in patients with these cancers. In the next step of our investigation, we will evaluate the influence of enzyme levels on the compounds cytotoxicity and cellular response induced by the studied drugs in breast and colon cancer cells. Therefore, we intend to predict the complicated role of individual enzyme levels in the patient response after antitumor drug treatment. To gain our goal we created systems of MCF-7 and HCT116 cells, each represented by model three cell lines—control cell line, containing empty vector (EV cells) and cell lines overexpressed with P4503A4 or UGT1A10 isoenzyme (CYP3A4 or UGT1A10 cells). We decided to work on transfected cell lines as they are overexpressed with one particular isoenzyme what can provide more specified and clear conclusions.

## 2. Results and Discussion

### 2.1. P4503A4 and UGT Expression in MCF-7 and HCT116 Cells

**Figure 2:**
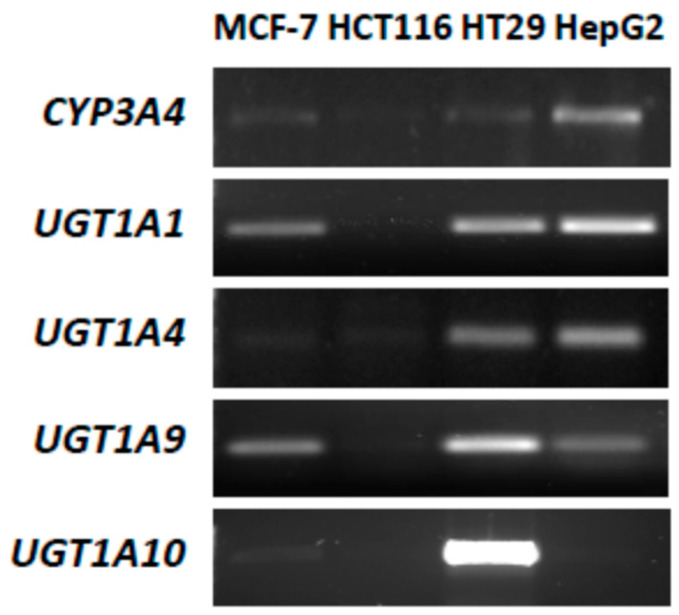
Reverse transcription-polymerase chain reaction (RT-PCR) analysis of mRNA expression of P4503A4 isoenzyme and selected UDP-glucuronosyltranspherase (UGT) isoenzymes: 1A1, 1A4, 1A9 and 1A10 in control, untreated MCF-7, HCT116, HT29 and HepG2 cells.

P4503A4 and UGT isoenzyme expression and their enzymatic activity were evaluated in the studied MCF-7 and HCT116 cells and, for comparison, in HepG2 cells well characterized with respect to metabolic potency. The HT29 cell line, with a naturally high level of UGT isoenzymes, particularly UGT1A10, was also included. Reverse transcription polymerase chain reaction (RT-PCR) (Figure 2) revealed that *CYP3A4* (gene name of cytochrome P4503A4) was expressed distinctly only in control HepG2 cells. The significant enzymatic activity of P4503A4 was also found in HepG2 cells (Table 1). In MCF-7 and HCT116 cells, the expression and activity of P4503A4 isoenzyme were insignificant. However, both cell lines obtained after stable overexpression with P4503A4 expressed higher enzymatic activity but still lower than in HepG2 cells. 

Next, the expression of selected UGT isoenzymes, *UGT1A1*, *UGT1A4*, *UGT1A9* and *UGT1A10*, in the four studied cell lines was examined. These UGT isoenzymes were considered because they participate in the glucuronidation of crucial natural substrates as bilirubin (*UGT1A1*) [42] or clinically used therapeutics (*UGT1A4*) [43] and specifically outside the liver in the metabolism of studied antitumor agents, compounds C-1305 and C-1311 (*UGT1A9*, *UGT1A10*) [33]. 

Results indicated that HCT116 cells did not express any of the studied UGT isoenzymes (Figure 2) and *O*-glucuronidation did not occur (Table 1), which is consistent with published data [44]. MCF-7 cells expressed *UGT1A1* and *UGT1A9* at low levels, whereas *UGT1A4* and UGT1A10 genes were not detected (Figure 2). The glucuronidation in these cells proceeded at a very limited rate (Table 1). HT29 cells stood out from the others with high levels of all studied UGT isoenzymes with predominantly intestinal UGT1A10. In contrast, the last isoenzyme was absent in the control HepG2 cell line, whereas the others were present. However, MCF-7 and HCT116 cell transfection with UGT1A10 resulted in a strong increase of this isoenzyme activity, particularly in HCT116 cells (Table 1). Moreover, UGT enzyme activity in transfected HCT116-UGT1A10 cells was higher than total activity of all UGT isoenzymes present in HepG2 cells but was still much lower compared to HT29 cells as described above.

### 2.2. Cytotoxic Effects of Studied Compounds against Cancer Cells

The cytotoxicity of C-1305 and C-1311 was evaluated in the panel of six cancer cell lines. There were three cell lines each of breast and colon cancer—one control with empty vector (EV) cells and two overexpressed with P4503A4 and UGT1A10 isoenzymes (CYP3A4 and UGT1A10 cells). Treatment of each cell line with 0.0001 to 100 µM of both compounds gave a concentration-dependent inhibition of cell proliferation, which resulted in the IC_50_ and IC_80_/IC_90_ values presented in Table 2. MCF-7 cells with empty vector expressed lower sensitivity in the presence of C-1305 than of C-1311, with IC_50_ equal to 1.87 ± 0.05 and 0.36 ± 0.08 µM, respectively, whereas HCT116-EV cells were similarly sensitive to both compounds, with IC_50_ near 1.0 µM and IC_90_ close to 10 µM.

Stable transfection of MCF-7 with *CYP3A4* isoenzyme led to higher sensitivity of transfected cells toward both C-1305 and C-1311 by 30% according to IC_50_ and IC_80_ values. Furthermore, the cytotoxic effect of C-1305 was also 30% higher against MCF-7-UGT1A10 cells than against MCF-7-EV. In contrast, the cytotoxicity of C-1311 was similar in the presence and absence of UGT1A10 isoenzyme in MCF-7 cells. Three cell lines of HCT116 gave similar IC_50_ and IC_80_ values for C-1305. Interestingly, the IC_90_ value calculated for HCT116-CYP3A4 cells treated with C-1305 was much higher than for HCT116-EV cells. The cytotoxicity results for C-1311 against HCT116 cells indicated that P4503A4 overexpression only slightly increased the drug effect, whereas higher levels of UGT1A10 resulted in significantly lower cytotoxicity of C-1311 against HCT116 cells (IC_50_ from 0.96 to 1.38 µM, IC_80_ from 5.37 to 9.31 µM, IC_90_ from 11.19 to 19.37 µM; Table 2). Thus, the possibility that C-1311 glucuronidation in HCT116-UGT1A10 cells would lead to lower drug activity against this cell line, which is consistent with the fact that glucuronidation usually decreases the activity of the drug [45,46].

### 2.3. Metabolic Transformation of C-1305 and C-1311 in MCF-7 and HCT116 Cells

**Figure 3:**
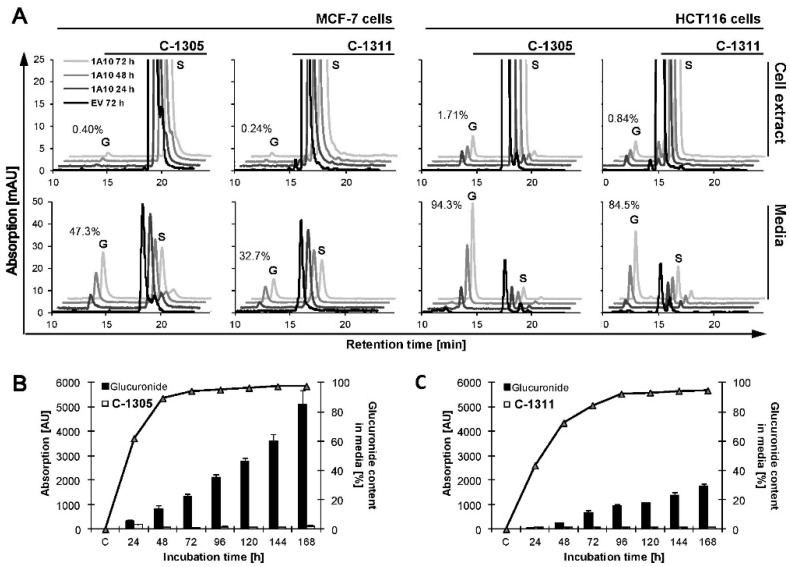
Metabolic transformation of C-1305 and C-1311 compounds in MCF-7 and HCT116 cells. (**A**) MCF-7-EV, MCF-7-UGT1A10, HCT116-EV and HCT116-UGT1A10 cells were treated with 30 mM C-1305 or C-1311 for 24, 48 or 72 h and methanol extract or media was subjected to high-performance liquid chromatography (HPLC) analysis as described in Materials and Methods. Peaks marked as S are substrates, C-1305 or C-1311, while G indicates glucuronide products of studied compounds. Numbers on chromatograms are percentage of glucuronide products formed after 72 h of incubation. (**B**,**C**) Percentage content (line (%)) and total amount expressed as absorption (bars (AU)) of glucuronide forms of (**B**) C-1305 and (**C**) C-1311 in media of HCT116-UGT1A10 cells treated with 30 mM drug for up to 168 h. Every day starting from 24 h of incubation 200 µL of media was taken and subjected to HPLC analysis.

C-1305 and C-1311 biotransformation was studied in MCF-7 and HCT116 cells with empty vector (EV) cells and cells stably transfected with *CYP3A4* and *UGT1A10* isoenzymes. The two compounds underwent very slight transformation in both control (EV) cell lines. Only one metabolite of C-1305 in cell extracts and culture media was observed. C-1311 metabolized to several products in cell extracts and one product in culture media but only in HCT116 cells (Figure 3A,B). Moreover, the metabolic profiles of C-1305 and C-1311 were not changed in MCF-7 and HCT116 cells after their overexpression with P4503A4 isoenzyme [47]. Thus, P4503A4 does not take part in the metabolism of studied acridinone derivatives, as we previously showed with recombinant isoenzymes in a non-cellular system [26,27] and in HepG2 cells [29,31].

In turn, MCF-7 and HCT116 cells transfected with UGT1A10 and incubated with both compounds (S) gave other results. High-performance liquid chromatography (HPLC) peaks (G) coming from C-1305 and C-1311 metabolites, which were collected after 24–72 h of incubation with 50 µM of the studied compounds, are presented in Figure 3A. We found in cell extracts and culture media products of metabolic transformation of studied compounds and electrospray ionization mass spectroscopy (ESI-MS) analysis confirmed that the new peak (G) observed on chromatograms obtained for HCT116-UGT1A10 cells represented glucuronide derivatives on the hydroxyl group of C-1305 and C-1311 [48].

The results in Figure 3A indicate that the levels of C-1305 and C-1311 glucuronides in cell extracts of MCF-7 and HCT116 cells overexpressing UGT1A10 enzyme were very low and only slightly increased to less than 2% for both compounds during 72 h incubation time. A particularly low level of UGT metabolites was observed in the extracts of MCF-7-UGT1A10 cells. Looking for the products of UGT-mediated metabolism after the incubation of studied compounds with tumor cells, we performed HPLC analysis not only of the cell extracts but also culture media. Figure 3A demonstrates that the content of glucuronide metabolites of C-1305 and C-1311 was 47% and 33%, respectively, in MCF-7 cells and 94% and 85%, respectively, in HCT116 cells. Thus, C-1305 compound underwent glucuronidation faster than C-1311 and the process was more effective in HCT116 than in MCF-7 cells. The demonstrated high level of glucuronide metabolites in cell media after drug incubation indicates that high amounts of C-1305 and C-1311 glucuronides were excreted outside the cell. The process of glucuronidation is considered to be a detoxification and excretive pathway, so a high level of glucuronide products observed in media is consistent with that concept. What is more, this is also why such a small concentration of glucuronides stayed inside the cell [43].

It is worth mentioning that the amount of glucuronides of C-1305 and C-1311 continuously increased in culture medium during prolonged incubation time (measured up to 168 h), as shown in Figure 3B,C. C-1305 underwent glucuronidation three times faster than C-1311. 

### 2.4. Modulation of P4503A4 and UGT1A10 Activity by C-1305 and C-1311

**Figure 4:**
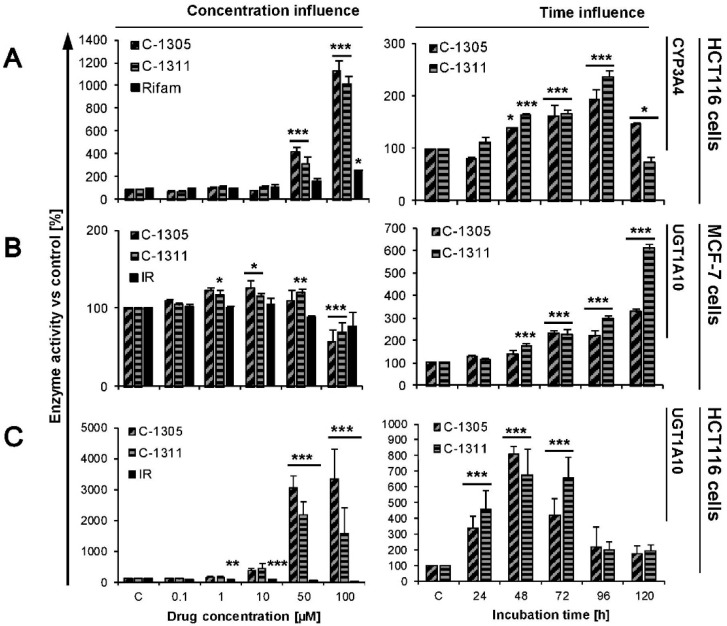
Activity of P4503A4 and UGT1A10 enzymes in MCF-7 and HCT116 cells. HCT116-CYP3A4, MCF-7-UGT1A10 and HCT116-UGT1A10 cells were treated with C-1305, C-1311 or irinotecan (IR)/rifampicin (Rifam) for 24 h with increasing concentrations of drugs (0.1, 1, 10, 50, 100 µM) or with 10 µM of C-1305 and C-1311 from 24 up to 120 h. After that, activity of UGT1A10 and P4503A4 was measured as described in Materials and Methods. Drug treatment concentration and time influence on activity of (**A**) P4503A4 in HCT116-CYP3A4, (**B**) UGT1A10 in MCF-7-UGT1A10 cells and (**C**) UGT1A10 in HCT116-UGT1A10 cells are presented on graphs, showing average of at least three experiments. Significant difference in analysis of variance (one-way ANOVA) of cell percentages between control, stably transfected with EV cells and UGT1A10- or CYP3A4-transfected are indicated as follows: * *p* ≤ 0.05; ** *p* ≤ 0.001, *** *p* ≤ 0.0001.

The impacts of C-1305 and C-1311 compounds on enzymatic activity of P4503A4 was investigated only in HCT116 cells, because we demonstrated too low activity of this enzyme in MCF-7 cells, even after stable transfection with *CYP3A4*. Our results (Figure 4A) indicate that HCT116-CYP3A4 cells were not sensitive to the modification of enzymatic activity by both compounds up to 10 µM concentration after 24 h incubation. However, in the presence of higher concentrations of drugs (up to 100 µM) catalytic capacity of P4503A4 strongly increased—12 times for C-1305 and 10 times for C-1311. It should be noted that this is a much higher increase compared to the two times increase for rifampicin, a well-known inducer of P4503A4 (Figure 4A) [49]. During long-term treatment of HCT116-CYP3A4 cells with C-1305 and C-1311, the activity of P4503A4 also gradually increased up to 96 h of incubation, then started to go down after 120 h. Concluding, we show that C-1305 and C-1311 are strong P4503A4 inducers. However, our previous studies in non-cellular systems revealed that C-1305 and C-1311 incubated with recombinant P4503A4 as its selective mechanism-based inactivator [25,26] but later in HepG2 cells showed that in living cells C-1305 increased activity of P4503A4 [50]. It suggests that the activation of P4503A4 catalytic properties does not result from direct interaction between the compounds and the P450 enzyme but through a cellular regulatory pathway and this activation is stronger than any direct drug inhibition features.

Figure 4B,C demonstrate the impact of the studied compounds on UGT1A10 activity in MCF-7-UGT1A10 and HCT116-1A10 cells. After 24 h of incubation, the increasing concentration of C-1305 and C-1311 up to 50 µM slightly changed UGT1A10 activity in MCF-7-UGT1A10 cells (Figure 4B). C-1311 caused an increase in the *O*-glucuronidation process at concentrations ranging from 1 to 50 µM and C-1305 only at 10 µM. Both compounds at 100 µM concentration decreased the activity of UGT1A10. At the same time, irinotecan did not affect UGT1A10 activity. On the other hand, time-dependent measurement of the impact of 10 µM drugs on UGT1A10 activity in MCF-7-UGT1A10 showed that C-1305 and C-1311 systematically and strongly induced enzymatic activity, by more than 3 times for C-1305 and 6 times for C-1311 (Figure 4B).

The strong induction of UGT1A10 by acridinones was also observed in HCT116-UGT1A10 cells (Figure 4C). Twenty-four-hour incubation with increasing concentrations of C-1305 and C-1311 showed that both compounds in HCT116-UGT1A10 cells firmly raised the enzyme capacity up to around 20 times for C-1305 and 30 times for C-1311 when higher concentrations of drugs were used (50 and 100 µM). Irinotecan in the same experiment inhibited the activity of UGT1A10 in HCT116-UGT1A10 cells, decreasing the capacity of the enzyme to 20% versus untreated control cells. Extended treatment of cells with 10 µM concentration of C-1305 and C-1311 also showed that both compounds strongly induced activity of UGT1A10 and the highest capacity of cells to promote glucuronidation was observed after 48 h and was 7–8 times higher than in control HCT116-UGT1A10 cells. After 72 h, the activity of UGT1A10 in studied cells started to decrease to 180% on average versus untreated cells. The induction of UGT1A10 isoenzyme by C-1305 and C-1311 was stronger in HCT116-UGT1A10 than MCF-7-UGT1A10 cells. Both compounds increased the enzyme activity in a similar way, whereas irinotecan led to inhibition of this enzyme. Our previous studies revealed that C-1305 and C-1311 did not modulate the activity of recombinant UGT1A19 and UGT1A10 isoenzymes and slightly influenced the activity of UGT1A1 [51]. However, in the same studies we showed that both compounds can indirectly change the enzymatic capacity of UGT enzymes in living cancer cells. Acridinones in HepG2 cells (lacking intestinal UGT1A10 isoform) increased the *O*-glucuronidation process, particularly in the case of C-1311. In HT29 cells, which exhibit extremely high expression and activity of nearly all isoforms of UGTs, especially UGT1A10, C-1311 caused a slight increase of UGT activity (at 10 and 50 µM) and at the highest concentration (100 µM) both compounds decreased the *O*-glucuronidation process. Concluding, acridinone derivatives can modulate the activity of some UGT enzymes but via an indirect pathway, as in case of P4503A4 modulation. What is interesting is that in all experiments conducted on different cancer cells, irinotecan turned to be the inhibitor of UGT enzymes.

### 2.5. Effects of C-1305 and C-1311 on Cell Cycle Progression of Control MCF-7-EV and HCT116-EV Cells

**Figure 5:**
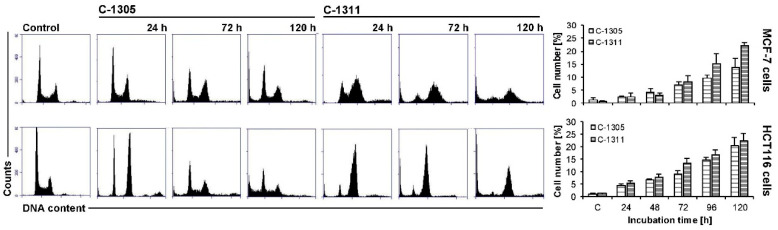
Cell cycle distribution of MCF-7-EV and HCT116-EV cells. Cells were untreated (control) or treated with IC_80_ dosage for MCF-7 and IC_90_ for HCT116 cells of C-1305 or C-1311 compounds for the times indicated and subjected to propidium iodide staining and flow cytometry as described in Materials and Methods. Histograms show number of cells (*y*-axis) versus DNA content (*x*-axis) and are representative of at least three experiments for each condition. Bar graphs show data quantitation with percentage of cells with less than 2N DNA (sub-G1 fraction).

The impact of C-1305 and C-1311 on cell cycle progression of breast and colon cancer cells was measured by flow cytometry. Cells were treated with drug concentration equal to IC_80_ for MCF-7 and IC_90_ for HCT116 cells for 0–120 h (Figure 5). Following treatment of MCF-7-EV cells with C-1305, the population of cells in G1 phase slightly decreased during the time of incubation, which was connected with an increased number of cells with degraded DNA (sub-G1 phase, typical of late-stage cell death), which reached around 14% after 120 h of treatment (Figure 5). Exposure of MCF-7-EV cells to C-1311 led to accumulation of cells in G2/M phase (60% after 48–72 h versus 25% in control cells) and after that this population started to decrease. Concomitantly with the mentioned changes, C-1311 led to an increased sub-G1 population, which reached 22% after 120 h of treatment (Figure 5). 

In HCT116-EV cells treated with C-1305, an accumulation of cells in G2/M phase was observed (increase from 26% to around 50% after 24 h of incubation; Figure 5). Concomitantly, the number of cells in G1 phase reduced from 57% in untreated cells to around 25–30% through the whole time of incubation. Interestingly, after 24 h of drug treatment, cells undergoing DNA synthesis amounted to only 2%, then a little bit more. Furthermore, the number of polyploid cells (with DNA more than 4N) significantly increased just after 24 h of incubation from 4.5% in control to nearly 20% after 72 h and then started to decrease. Such observations suggest that mitotic catastrophe can occur. Along with the time of incubation with C-1305, the population of cells in sub-G1 phase systematically increased up to 18% after 120 h. C-1311 in HCT116-EV caused a little more profound change in cell cycle progression than C-1305. Cells treated with C-1311 underwent accumulation in G2/M phase, which was the most noticeable after 24 h (71% compared to 26% in control; Figure 5). Simultaneously, the population of cells in G1 and S phase firmly decreased from the first hours of incubation. After 120 h of incubation, the number of cells in sub-G1 phase reached 23%. What is more, the population of polyploid cells was not so conspicuous in cells treated by C-1311 in comparison to C-1305. Concluding, both C-1305 and C-1311 caused similar changes in the cell cycle distribution of MCF-7-EV and HCT116-EV and these changes were more intense in HCT116-EV than in MCF-7-EV cells.

### 2.6. Induction of Cell Death in MCF-7-EV and HCT116-EV Cells

**Figure 6:**
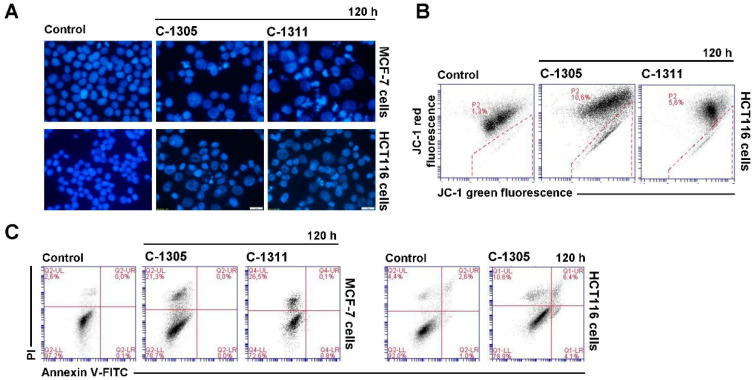
Cellular response of MCF-7-EV and HCT116-EV cells. (**A**) Representative pictures of changes in nuclear morphology of MCF-7 and HCT116 cells treated with IC_80_ or IC_90_ dosage, respectively, of C-1305 and C-1311 compounds. Cells were stained with 4′,6-diamidino-2-phenylindole (DAPI; 1 mg/mL) and visualized under fluorescent microscope (400× magnification); (**B**) Cytometric analysis of changes in mitochondrial transmembrane potential (ΔΨm) in MCF-7-EV and HCT116-EV cells treated with drugs for 120 h and labeled with JC-1 dye. Presented cytograms are representative of three independent experiments. Marked gates are populations of cells with decreased mitochondrial transmembrane potential (ΔΨm) (green fluorescence); (**C**) Phosphatidylserine externalization and membrane disruption in MCF-7-EV and HCT116-EV cells after 120 h of treatment with C-1305 and C-1311. Representative bivariate flow cytometry histograms of annexin V–fluorescein isothiocyanate (FITC) signal versus PI signal are shown. Bottom left quadrant represents live cells (annexin V negative, PI negative); bottom right quadrant represents early apoptotic cells (annexin V positive, PI negative); top right quadrant represents late apoptotic cells (annexin V positive, PI positive); top left quadrant represents primary necrotic cells (annexin V negative, PI positive).

Cell cycle analysis revealed that in MCF-7 and HCT116 cells treated with acridinone derivatives, a considerable sub-G1 population occurred, indicating apoptosis or another process leading to DNA degradation. It has to be mentioned that MCF-7 cells lack caspase-3 [52], one of the main enzymes essential for activation of apoptosis [53]. Thus, this programmed cell death should not be induced in MCF-7 cells. Fluorescent microscopy observation of cell nucleus morphology, analysis of translocation of phosphatidylserine and disruption of cell membrane as well as decreased mitochondrial membrane potential were used to determine the type of cell death (Figure 6). Changes in nucleus morphology triggered by both acridinones were similar in MCF-7-EV, although were more profound in cells treated with C-1311. Most of the observed changes were fragmentation and condensation of chromatin and increased number of cells with altered nucleus morphology during the time of incubation (Figure 6A). Typical apoptotic bodies were not found during the observation of MCF-7-EV cells. However, pyknotic nucleus (dense and compact), which can appear during apoptosis, was present but also necrosis [54], many mitotic figures and some multinucleated cells characteristic of mitotic catastrophe were also visible. Many changes were difficult to classify. The nuclei of many MCF-7-EV cells exposed to acridinones were enlarged, which can indicate necrosis. Evaluation of phosphatidylserine externalization and disruption of cell membrane (annexin V/propidium iodide (PI) test) confirmed that apoptosis was not induced in MCF-7-EV cells exposed to C-1305 and C-1311. The process of phosphatidylserine translocation from internal to external leaflet was not noticeable (no cells were annexin-V–fluorescein isothiocyanate (FITC) positive; Figure 6C). Flow cytometry analysis of caspase-3 activation revealed that the enzyme was not induced [55]. During the time of incubation with both acridinones, cell membrane perturbation was observed in MCF-7-EV cells (PI positive) and the process referred to 18% and 19% cells treated with C-1305 and C-1311, respectively, after 120 h (Figure 6A).

The morphology of nuclei of HCT116-EV cells treated with acridinone derivatives changed with the time of treatment, mainly for those exposed to C-1305 (Figure 6A). Microscopic observations revealed that C-1305-treated HCT116-EV cells showed changes characteristic of apoptosis, such as condensation and subsequent fragmentation of chromatin, with transitional distribution in the nuclear periphery and formation of typical apoptotic bodies. These changes started to appear after 72 h of incubation. Some of the cells were multinucleated, which is indicative of mitotic catastrophe. Many nuclei were enlarged, which in turn can be evidence of necrosis (Figure 6). Induction of apoptosis in HCT116-EV treated with C-1305 was confirmed by analysis of decreased mitochondrial membrane potential and phosphatidylserine externalization (Figure 6B,C). The population with green fluorescence JC-1 was 12%, annexin V-FITC/PI positive cells were around 10% after 120 h of C-1305 treatment and necrotic cells, PI positive (and annexin V-FITC negative) were 11.5% at the end of the time of incubation (120 h) (Figure 6C).

HCT116-EV cells exposed to C-1311 revealed fewer changes in the morphology of nuclei than those treated with C-1305 (Figure 6A). Some of the cells showed features characteristic of apoptosis (mainly pyknotic cells) and mitotic catastrophe (multinucleated cells). Decreased mitochondrial membrane potential was observed in 5% after 120 h, confirming the presence of apoptotic cell death in a small population (Figure 6B). Analysis of phosphatidylserine externalization could not be performed for C-1311 because this derivative itself exhibits fluorescence that obscures the signal and disturbs correct reading. Summing up, HCT116-EV cells were more sensitive to C-1305 compound than C-1311, contrary to the results obtained for MCF-7-EV cells, where both compounds exhibited rather similar effects against breast cancer cells.

### 2.7. P4503A4 Influence on Cellular Response Induced by C-1305 and C-1311 in MCF-7 and HCT116 Cells

As shown above, both studied drugs, C-1305 and C-1311, induced P4503A4 enzyme activity in HCT116 cells. A similar effect could be seen in HepG2 cells, where C-1305 enhanced expression, protein level and activity of P4503A4 and these processes were PXR-independent [50]. This enzyme takes part in the metabolism of endogenous compounds and may be indirectly responsible for cell proliferation [39]. It also enhanced the risk of breast cancer development [56,57] and drug resistance [58]. Therefore, we decided to test the influence of higher P4503A4 activity on cellular response in acridinone-treated MCF-7 and HCT116 cells. We developed cell lines with stable overexpression of P4503A4 that exhibited increased expression of P4503A4 by around 9 and 11 times in MCF-7 and HCT116 cells, respectively (real-time PCR; [59]) and enhanced enzyme activity, increased by 3.5 and 8.6 times for MCF-7 and HCT116 cells, respectively (Table 1).

**Figure 7:**
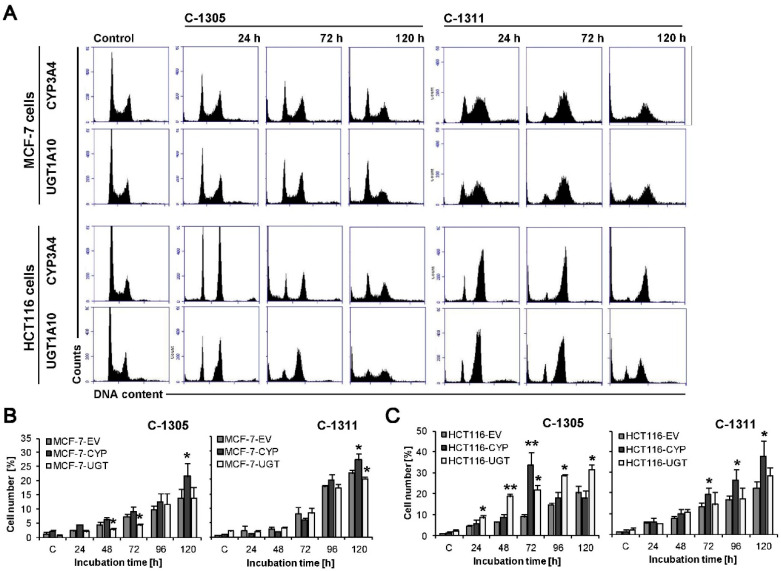
Cell cycle distribution of MCF-7-CYP3A4, MCF-7-UGT1A10, HCT116-CYP3A4 and HCT116-UGT1A10 cells. Cells were untreated (control) or treated with C-1305 or C-1311 (IC_80_ for MCF-7 cells, IC_90_ for HCT116) for the times indicated and subjected to propidium iodide staining and flow cytometry as described in Materials and Methods. (**A**) Histograms show number of cells (*y*-axis) versus DNA content (*x*-axis) and are representative of at least three experiments for each condition. (**B**,**C**) Bar graphs show data quantitation with percentage of cells with less than 2N DNA (sub-G1 fraction): three types of MCF-7 (**B**) and HCT116 (**C**) cells treated with C-1305 or C-1311. Results are expressed as mean ± SEM (*n* ≥ 3). Significant differences in cell percentages between control, stably transfected with EV cells and UGT1A10- or CYP3A4-transfected are indicated as follows: * *p* ≤ 0.05; ** *p* ≤ 0.001.

**Figure 8:**
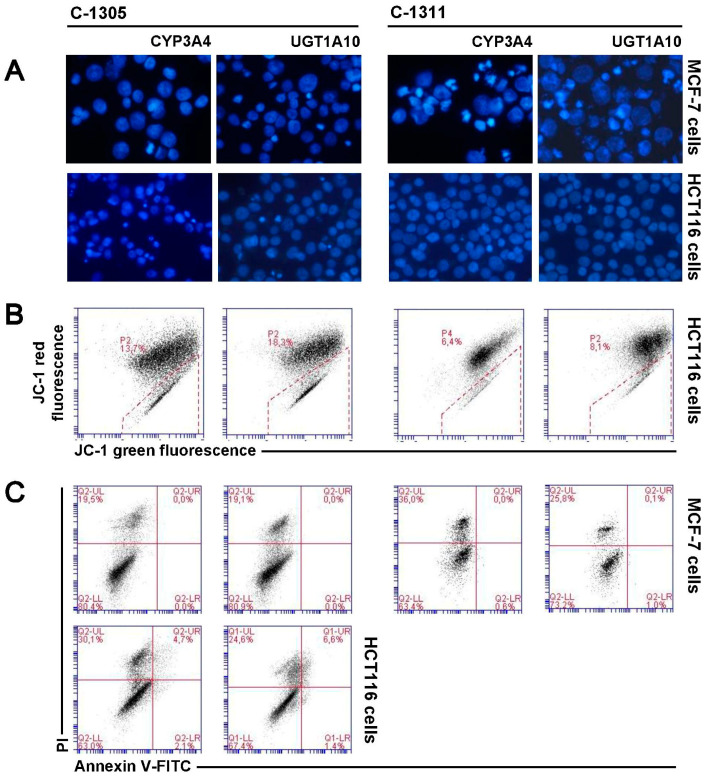
Cellular response of MCF-7-CYP3A4, MCF-7-UGT1A10, HCT116-CYP3A4 and HCT116-UGT1A10 cells to 120 h of C-1305 and C-1311 treatment. (**A**) Representative analysis of changes in nuclear morphology of two types of MCF-7 and HCT116 cells exposed to C-1305 and C-1311 (IC_80_ for MCF-7, IC_90_ for HCT116). Cells were stained with DAPI (1 mg/mL) and visualized under fluorescent microscope (400× magnification); (**B**) Cytometric analysis of changes in mitochondrial transmembrane potential (ΔΨm) in two types of HCT116 cells treated with both compounds and labeled with JC-1 dye. Cytograms are representative of three independent experiments. Marked gates are populations of cells with decreased mitochondrial transmembrane potential (ΔΨm) (green fluorescence); (**C**) Phosphatidylserine externalization and membrane disruption in two types of MCF-7 and HCT116 cells after 120 h of treatment with C-1305 and C-1311. Representative bivariate flow cytometry histograms of annexin V-FITC signal versus PI signal. Bottom left quadrant represents live cells (annexin V negative, PI negative); bottom right quadrant represents early apoptotic cells (annexin V positive, PI negative); top right quadrant represents late apoptotic cells (annexin V positive, PI positive); top left quadrant represents primary necrotic cells (annexin V negative, PI positive). Annexin V/PI test could not be performed for HCT116 cells because of too high autofluorescence of 10 µM C-1311.

**Figure 9:**
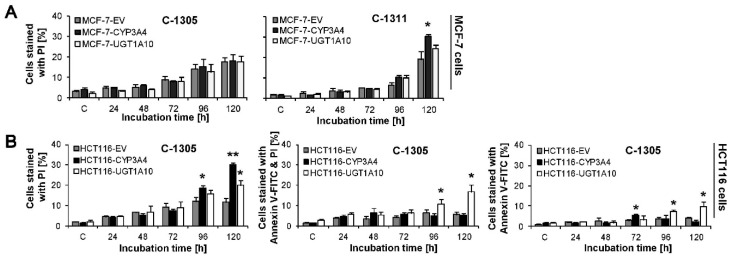
Quantitative analysis of phosphatidylserine externalization and membrane disruption in MCF-7 and HCT116 cells after 120 h of treatment with C-1305 and C-1311 at IC_90_. (**A**) Three types of PI-positive stained MCF-7 cells after 120 h of C-1305 and C-1311 treatment; (**B**) Populations of cells from individual quadrants: annexin V positive or annexin V and PI positive or PI positive, obtained from annexin V/PI test conducted for three types of MCF-7 and HCT116 cells exposed to C-1305 and C-1311 at IC_90_. Significant differences in cell percentages between control, stably transfected with EV cells and UGT1A10- or CYP3A4-transfected are indicated as follows: * *p* ≤ 0.05; ** *p* ≤ 0.001.

Cytotoxicity studies revealed that MCF-7-CYP3A4 cells were more sensitive to C-1305 and C-1311 compounds than control MCF-7-EV cells (Table 2). However, the distribution of MCF-7-CYP3A4 cells in each phase of the cell cycle treated with C-1305 was similar to that observed in control EV cells. Only the population of cells in sub-G1 phase with degraded DNA significantly increased after 120 h of drug treatment in cells overexpressing P4503A4 and reached 23%, compared to 14% in EV cells (Figure 7A,B). There was no remarkable difference in morphology of cell nuclei and membrane perturbation between MCF-7-CYP3A4 and EV cells exposed to C-1305 (Figure 8A,C). Thus, P4503A4 overexpression had an impact on cell proliferation and DNA degradation in C-1305-treated MCF-7 cells but did not increase the cell population undergoing necrosis (Figure 8C and Figure 9). 

The C-1311 compound did not change the profiles on histograms obtained in cell cycle distribution analysis of MCF-7-CYP3A4 cells in comparison to MCF-7-EV cells (Figure 5A and Figure 7A). However, the population of cells with less than 2N DNA increased significantly after 120 h, from 22% in EV cells to 27% in CYP3A4 cells. Greater degradation of DNA resulted in more profound changes in morphology of nuclei of MCF-7-CYP3A4 cells treated with C-1311 (more necrotic, mitotic and multinucleated cells), which was observed from 72 h (Figure 8A). C-1311 in MCF-7-CYP3A4 cells also caused more noticeable perturbations in cell membrane than in MCF-7-EV cells (starting from 96 h). There were 30% PI-positive cells after 120 h of incubation (19% in EV cells). Summarizing, higher activity of P4503A4 isoenzyme caused stronger cellular effects in MCF-7 cells triggered by both acridinone derivatives, C-1305 and C-1311 but more intensely by imidazoacridinone (C-1311). We previously observed altered cellular response to C-1311 treatment under conditions of overexpressed P4503A4 in CHO cells [32] and HepG2 cells [31], where this enzyme did not participate in imidazoacridinone biotransformation. Usually P4503A4 changes the rate of cell death with drugs that are metabolized by this enzyme, such as tamoxifen [60,61], docetaxel [62], paclitaxel [63], vinca alkaloids [64], imatinib [65] and many more [66]. However, recent reports show that P4503A4 takes part in arachidonic acid metabolism and the biosynthesis of epoxyeicosatrienoic acids (EETs), which are implicated in tumor growth, metastasis and angiogenesis. The influence of EETs on cancer progression was shown in models of human breast cancer [39,55] with different expression of P4503A4. Thus, the altered cellular response of MCF-7 cells overexpressing P4503A4 observed after C-1305 and C-1311 treatment is suggested to result from enzyme-mediated endogenous metabolism. 

The 72 h analysis of cytotoxicity of C-1305 and C-1311 against HCT116 panel cells revealed no difference in IC_50_ and IC_80_ values between EV cells and those overexpressing P4503A4. Interestingly, IC_90_ value calculated for C-1305 against HCT116-CYP3A4 cells was much higher than that for EV cells. The increase from 10 µM to 26 µM (Table 2) suggests that P4503A4 overexpression makes HCT116 cells less sensitive to triazoloacridinone derivative. However, the study on cellular response triggered by C-1305 in HCT116-CYP3A4 showed the opposite effect when increasing the enzyme’s activity. Cell cycle analysis (Figure 7A) revealed that after 72 h of cell incubation with C-1305, the number of cells with degraded DNA very strongly increased, reaching 28.5% (9% in EV cells) and then decreased to a similar level as in EV cells (Figure 7C). Concomitantly, the population of polyploid cells was smaller in cells overexpressing P4503A4 isoenzyme. Furthermore, DAPI staining of nuclei showed that in HCT116-CYP3A4 cells, C-1305 strongly increased the number of cells with abnormal morphology of nuclei (mitotic and apoptotic cells) in comparison to EV cells (Figure 8A). Similarly, more HCT116 cells were annexin V-FITC positive (Figure 8C) and exhibited lower mitochondrial transmembrane potential (Figure 8B) when P4503A4 was overexpressed. What is more, the population of PI-positive cells in HCT116-CYP3A4 cells significantly increased in comparison to EV cells and after 120 h 30% of cells lost membrane integrity (11.5% in EV cells; Figure 9B). The described results showed that high activity of P4503A4 isoenzyme in C-1305-treated HCT116 cells can temporarily lead to DNA degradation in a greater population of cells and subsequently to the induction of apoptosis and necrosis to a greater extent. 

In light of the above findings, we decided to carry out the cytotoxicity examination after shorter and longer incubation times—24 and 120 h. The cytotoxic effect of C-1305 against HCT116-CYP3A4 cells after 24 h of treatment was much stronger than against EV cells—all determined IC values decreased by nearly 2 times. However, 120 h of incubation resulted in increased IC values by even more than 2 times in HCT116-CYP3A4 cells in comparison to EV cells. Thus, overexpression of P4503A4 isoenzyme makes HCT116 cells in the first days of exposure to C-1305 more sensitive and susceptible to cell death but after prolonged drug treatment time, the high activity of the enzyme can have a protective effect on the cells.

The effect of high P4503A4 activity against HCT116 cells treated with C-1311 was not so potent against C-1305-treated cells. The 24 h cytotoxic assay revealed that HCT116-CYP3A4 cells were more sensitive to C-1311 than EV cells. However, 72 h of incubation showed that overexpression of P4503A4 did not influence the sensitivity of HCT116 cells exposed to C-1311, while during prolonged incubation cells appeared to be less sensitive. Degradation of DNA triggered by C-1311 was more profound when P4503A4 isoenzyme was overexpressed, although the profile of cell cycle phase distribution did not differ between CYP3A4 and EV cells. After 120 h, the number of HCT116-CYP3A4 cells in sub-G1 fraction reached 32% (23% in EV cells; Figure 7C). The changes in morphology of nuclei and the population of cells with decreased mitochondrial transmembrane potential increased after prolonged C-1311 treatment in HCT116-CYP3A4 cells in comparison to EV cells (Figure 6B and Figure 8B).

Concluding the above results, P4503A4 significantly altered the HCT116 response to C-1305 and C-1311 and this process seems to be independent of the drugs’ biotransformation and probably results from the indirect influence of P4503A4 on endogenous metabolism. 

### 2.8. UGT1A10 Isoenzyme Influence on Cellular Response Induced by C-1305 and C-1311 in MCF-7 and HCT116 Cells

We showed in our previous studies that under conditions of transiently enhanced activity of UGT1A10, which allows for glucuronidation of acridinone derivatives, the cytotoxicity and capacity for DNA degradation of C-1305 and C-1311 compounds were altered in KB-3 cells [34]. Therefore we decided to obtain cells with stable overexpression of UGT1A10 enzyme in MCF-7 and HCT116 cells. The study on cytotoxic effect triggered by C-1305 in MCF-7 cells revealed that cells overexpressing UGT1A10 were more sensitive to the drug than EV cells and IC_50_, IC_80_ and IC_90_ values decreased by around 20%, 30% and 40%, respectively (Table 2), which is an unusual effect, since glucuronidation is a process that typically leads to drug deactivation. This unique effect was also obtained for C-1305 toward the previously mentioned KB-3 cells [34]. On the other hand, the analysis of cell cycle distribution, nucleus morphology and membrane perturbation did not show any differences in cellular response between cell lines (Figure 5, Figure 6, Figure 7, Figure 8 and Figure 9). In turn, C-1311 after glucuronidation did not change its cytotoxicity against MCF-7 cells in comparison to native drug (Table 2). Moreover, cellular response triggered by imidazoacridinone was almost identical in MCF-7-UGT1A10 and EV cells. Such an effect is strongly desirable, since both compounds did not lose their ability to kill cancer cells after glucuronidation. 

The cytotoxicity of C-1305 determined after 24 and 72 h of treatment against HCT116-UGT1A10 was similar to that against EV cells. Only prolonged analysis of the cytotoxic effect of C-1305 (120 h) revealed that C-1305 undergoing glucuronidation is slightly less cytotoxic than the parental drug at high concentrations, while IC_50_ values were the same for both cell lines (Table 2). However, analysis of cell cycle distribution showed that C-1305 caused stronger degradation of DNA in HCT116-UGT1A10 than in EV cells—after 120 h, the sub-G1 fraction reached 31.5% (20.5% in EV cells) (Figure 6C). Microscopic observation showed that during the time of incubation the number of HCT116-UGT1A10 cells with apoptotic and mitotic features of nuclei also increased, much more than in EV cells. Also, phosphatidylserine externalization and membrane perturbation affected a larger population of HCT116 cells when C-1305 was glucuronidated. After 120 h of treatment, early apoptotic cells were 7.5% (4% in EV cells) and the combined population of late apoptotic and necrotic cells (PI-positive cells) was 36.5% (only 17% in EV cells; Figure 9B). The decrease in mitochondrial transmembrane potential occurred in 18% of HCT116-UGT1A10 cells after 120 h (13% in EV cells; Figure 8B). Thus, the induction of apoptosis and necrosis in HCT116 cells was much more potent when C-1305 was undergoing glucuronidation. For the first time we show strong evidence that glucuronidation of C-1305 significantly modulated its mechanism of action and enhanced its ability to kill more cells than in the case of native drug.

Cytotoxicity analysis of the second compound, C-1311, against HCT116-UGT1A10 cells showed that this drug was less active than against EV cells. Nearly all determined IC values after 72 and 120 h of treatment were higher than the corresponding values calculated for HCT116-EV cells. However, the lower proliferation inhibition of HCT116-UGT1A10 cells by C-1311 compound did not alter the cellular response induced by the drug in comparison to EV cells. C-1311 undergoing glucuronidation caused DNA degradation, changes in cell nuclei and mitochondrial potential drop at the same level as the parent drug (Figure 6, Figure 7, Figure 8 and Figure 9). As in MCF-7 cells, C-1311 maintained its pro-apoptotic and pro-necrotic properties after conjugation with glucuronic acid, also in HCT116 cells overexpressed with UGT1A10.

## 3. Materials and Methods

### 3.1. Chemicals and Reagents

5-Dimethylaminopropyloamino-8-hydroxytriazoloacridinone, C-1305 and 5-diethylaminoethylamino-8-hydroxyimidazoacridinone (Symadex), C-1311 were synthesized as monochlorides in the Department of Pharmaceutical Technology and Biochemistry, Gdańsk University of Technology, according to a previously published procedure [4,6]. Working solution of the drugs in 50% ethanol was freshly prepared just before use. Plasmids—pcDNA3.1 and pMONO-GFP were from Invitrogen (Carlsbad, CA, USA), pcDNA3/CYP3A4/c-myc/His was a gift from Prof. Peter Espenshade from John Hopkins University School of Medicine, Baltimore, MD, USA and pcDNA3.39/UGT1A10 [66] was a gift from Prof. Anna Radomińska-Pandya from University of Arkansas for Medical Science, Little Rock, AR, USA. Fetal bovine serum (FBS) was purchased from Biowest (Nuaille, France). G418 Solution was obtained from Roche (Basel, Switzerland). Plasmid Midi AX isolation Kit was purchased from AA Biotechnology (Gdynia, Poland). Maxima Hot Start Green PCR Master Mix and GeneRuler 50 bp DNA Ladder were obtained from Thermo Scientific (Waltham, MA, USA). Starters for PCR reaction were purchased from Genomed (Warsaw, Poland). Methanol and acetonitrile, both of HPLC grade, were purchased from Merck (Darmstadt, Germany). PI/RNase Staining Buffer, FITC Annexin V Apoptosis Detection Kit and Mitochondrial Membrane Potential Detection JC-1 Kit were obtained from BD Biosciences (San Diego, CA, USA). Protein Assay Dye Reagent Concentrate was obtained from Bio-Rad (Hercules, CA, USA). The following reagents were purchased from Sigma Aldrich (Merck) (Darmstadt, Germany)—3-(4,5-Dimethylthiazol-2-yl)-2,5-diphenyltetrazolium bromide (MTT), 4′,6-diamidine-2′-phenylindole (DAPI), 7-hydroxy-4-trifluoromethylcoumarin (7-OH-TFC), agarose, ammonium formate, dimethyl sulfoxide (DMSO), ethidium bromide, ethylenediaminetetraacetic acid (EDTA), formic acid, irinotecan, magnesium chloride, McCoy 5A medium, MEM medium, Nonidet P-40, penicillin-streptomycin solution, phenylmethanesulfonyl fluoride (PMSF), potassium chloride, potassium phosphate dibasic, potassium phosphatemonobasic, rifampicin, RPMI 1640 medium, sodium deoxycholate, sodium dodecyl sulfate (SDS), sodium, sodium phosphatedibasic, testosterone, Tris base, Tris hydrochloride, Trypsin-EDTA solution. The following reagents were purchased from POCH S.A. (Gliwice, Poland)—ethanol, methanol, sodium chloride, sodium fluoride. Ultra-pure deionized water R >18 MΩ·cm^−1^ was prepared by Milli-Q Integral Water Purification System, Merck Millipore (Billerica, MA, USA).

### 3.2. Cell Lines, Cultures and Creation of Stable Cell Lines

Human breast cancer cell line MCF-7, human colorectal carcinoma cell lines HCT116 and HT29 and human hepatoma cell line HepG2 were purchased from American Type Culture Collection (ATCC, Manassas, VA, USA). All cell lines were tested negatively for mycoplasma using Universal Mycoplasma Detection Kit, ATCC-30-1012K (ATCC). MCF-7 cells were maintained in RPMI 1640 medium, HCT116 and HT29 cells in McCoy 5A medium and HepG2 cells in MEM medium, all supplemented with 10% fetal bovine serum (FBS), 100 U/mL penicillin and 100 mg/mL streptomycin. Cells were grown in monolayers at 37 °C under 5% CO_2_. To create cell lines with stable overexpression of P4503A4 and UGT1A10 isoenzymes 1 × 10^6^ MCF-7 and HCT116 cells were transfected with 2 µg of pcDNA3/CYP3A4/c-myc/His or pcDNA3.39/UGT1A10 plasmid by nucleofection (Nucleofector 2b device, Lonza, Basel, Switzerland) in 100 µL of Amaxa Nucleofector Solution V (Lonza, Basel, Switzerland) according to the manufacturer’s protocol. The control cell line was developed by transfection of MCF-7 and HCT116 cells with empty vector, pcDNA3.1. The efficiency of nucleofection was monitored by green fluorescence protein (GFP) under fluorescence microscope. Isolation of single clones of the stable transfectants was accomplished by adding geneticin (G418, Roche, Basel, Switzerland) at a concentration of 400 µg/mL. Clones with the highest overexpression of P4503A4 and UGT1A10 were chosen by checking the activity of enzymes in each isolated clone. 

### 3.3. Cell Growth Inhibition Assay

To estimate cell viability, an MTT assay was used. MCF-7 and HCT116 cells (2000/well and 1000/well, respectively) were seeded in 96-well plates and the following day C-1305 or C-1311 was added at concentrations up to 100 μM. Stock solutions of acridinone compounds were prepared as 10 mM in 50% ethanol, reference drugs and enzymatic substrates in DMSO, with dilution in 50% ethanol. Controls received vehicle alone (final concentration of ethanol was 0.5%). After 24, 72 or 120 h, 3-(4,5-dimethylthiazol-2-yl)-2,5-diphenyltetrazolium bromide (MTT; 50 μg/well) was added for 3 h, followed by DMSO solubilization of the cells and absorbance reading at 540 nm. Each point was conducted at least 4 times and data are expressed relative to vehicle-treated controls. The concentration of the drug required to inhibit cells growth by 50% (IC_50_), 80% (IC_80_) and 90% (IC_90_) compared with untreated control cells was determined from the curves plotting survival as a function of dose. 

### 3.4. mRNA Isolation and Reverse Transcription Polymerase Chain Reaction (RT-PCR)

The relative expression of CYP3A4 and UGT1A10 isoenzymes in MCF-7 and HCT116 cells, wild type and transfected and HT29 and HepG2 cells was determined by reverse transcription polymerase chain reaction (RT-PCR). mRNA was isolated from 1 × 10^6^ confluent untreated cells using High Pure RNA Isolation Kit according to the manufacturer’s instructions (Roche, Basel, Switzerland). The concentration of RNA was determined by NanoDrop 2000 (Thermo Scientific, Pittsburgh, PA, USA). Then 1 μg of RNA was reverse transcribed using Transcriptor First Strand cDNA Synthesis Kit according to the manufacturer’s instructions (Roche Diagnostics, Mannheim, Germany) in 20 μL of reaction. The reverse transcription reaction was carried out for 30 min at 55 °C and stopped by heating to 85 °C for 5 min and placing on ice. cDNA samples obtained from all studied cells were subjected to PCR reaction in thermal cycler (Thermo Scientific, Eppendorf, Pittsburgh, PA, USA) with the following conditions—1 cycle for 4 min at 95 °C (preincubation); 45 cycles of amplification—30 s at 95 °C (denaturation), 30 s at 61 °C (annealing), 30 s at 72 °C (extension), 1 cycle for 5 min at 72 °C (final extension) and 1 cycle for 30 s at 4 °C (cooling). Primers were ordered from Genomed (Warsaw, Poland) and the sequences were as follows—CYP3A4 forward primer, TTTCCACCACCCCCAGTTAG; CYP3A4 reverse primer, CCACGCCAACAGTGATTACA; UGT1A1 forward primer, CCTTGCCTCAGAATTCCTTC; UGT1A1 reverse primer, ATTGATCCCAAAGAGAAAACCAC; UGT1A4 forward primer, ACGCTGGGCTACACTCAAGG; UGT1A4 reverse primer, TCATTATGCAGTAGCTCCACACA; UGT1A9 forward primer, GAGGAACATTTATTATGCCACCG; UGT1A9 reverse primer, GCAACAACCAAATTGATGTGTG; UGT1A10 forward primer, CCTCTTTCCTATGTCCCCAATGA; UGT1A10 reverse primer, CCTTAGTCTCCATGCGCTT TGC. Equal amounts of aliquots of PCR products were separated on 1.8% agarose gel and visualized by ethidium bromide staining. 

### 3.5. P4503A4 and UGT Activity Modulation in Living Cells

MCF-7 and HCT116 cell lines were treated with C-1305, C-1311, irinotecan or rifampicin for 24 h with different concentrations of drugs (0.1, 1, 10, 50, 100 µM) or with one concentration, 10 µM, for 24, 48, 72, 96 or 120 h. Studied acridinone compounds were prepared as 10 mM stock solutions in 50% ethanol, other drugs as 20 mM in DMSO and diluted in 50% ethanol, with the final concentration of alcohol at no more than 0.5%. After treatment, the culture medium was replaced with fresh medium containing 20 µM testosterone or 50 µM 7-OH-TFC, standard substrates of CYP3A4 isoenzyme or the *O*-glucuronidation process, respectively. All cells were incubated with CYP3A4 substrate for 24 h, while treatment with 7-OH-TFC lasted 2, 6 or 18 h for HT29, HCT116-UGT1A10 and MCF-7-UGT1A10 cells, respectively. Testosterone, 7-OH-TFC and their metabolic products were extracted from the media with acetonitrile (1:1, *v*/*v*), centrifuged (3 × 6 min, 14,000 rpm, 4 °C) and the supernatant was analyzed by HPLC (point 3.7). Cells remaining on the Petri dishes were scraped in ice-cold PBS, centrifuged (5 min, 1000 rpm, 4 °C), washed again and resuspended in RIPA solution. The cells were lysed on ice for 20 min, centrifuged (15 min, 14,000 rpm, 4 °C) and the protein concentration was determined using the Bradford assay (Bio-Rad, Hercules, CA, USA). CYP3A4 and UGT activity was calculated given both the cell viability expressed by protein concentration and detection of 6β-hydroxytestosterone and 7-OH-TFC glucuronide, respectively, by HPLC. For each point the experiment was done at least 4 times. 

### 3.6. Biotransformation of C-1305 and C-1311 in Alive Cells

Confluent MCF-7 and HCT116 cells were treated with studied compounds (30 mM) for 24, 48 or 72 h. After the treatment, 1 mL of media was taken for further analysis. The cells were scraped, washed twice with ice-cold PBS and centrifuged (1000 rpm, 5 min, 4 °C). The pellet was resuspended in 200 µL of 60% methanol. The cell-methanol mixture was sonicated in an ultrasonic bath for 15 min, placed in ice for 1 h, centrifuged (14,000 rpm, 20 min, 4 °C) and analyzed by HPLC (point 4.6). Drugs and their metabolites were extracted from the media with acetonitrile (1:1, *v*/*v*) and centrifuged (3 × 14,000 rpm, 6 min, 4 °C) and the supernatant was analyzed by HPLC. LC-MS/MS analysis of the products was carried out by electrospray ionization with positive ion detection using a Shimadzu LCMS-2020 system (Shimadzu Corp., Kyoto, Japan). 

### 3.7. High-Performance Liquid Chromatography (HPLC) Analysis

All metabolic transformations were analyzed using RP-HPLC with UV-vis detection with 5 mm Suplex pKb-100 analytical column (0.46 cm × 25 cm, C18) (Supelco, Bellefonte, PA, USA) with Waters HPLC system equipped with 1525 binary pump, 7725i Rheodyne injector, 2487 dual λ absorbance detector and 717 plus autosampler controlled with Breeze software (Waters Co., Milford, MA, USA). Aliquots of 200 µL of the prepared samples were analyzed by HPLC-UV-vis at a flow rate of 1 mL/min in 50 mM ammonium formate buffer, pH = 3.2, with a linear gradient from 15% to 80% methanol for 25 min, followed by a linear gradient from 80% to 100% for 3 min. The column was then re-equilibrated at initial conditions for 20 min between runs. The elution of sample components was monitored at 254 nm (testosterone transformation), 330 nm (7-OH-TFC glucuronidation) or 420 nm (C-1305 and C-1311 metabolism). 

### 3.8. Cell Cycle Analysis

MCF-7 and HCT116 cells were exposed to C-1305 and C-1311 at IC_80_ or IC_90_ dose, respectively, for 24 to 120 h. After drug treatment, trypsinized and floating cells (2 × 10^6^) were pooled, washed twice with ice-cold PBS and fixed in 70% (*v*/*v*) ethanol overnight at −20 °C. Cells were stained with propidium iodide (PI) (BD Biosciences, San Jose, CA, USA) and monitored by FACS Accuri C6 (BD Biosciences, San Jose, CA, USA).

### 3.9. Assessment of Cell Morphology

Nuclear morphology was examined under a fluorescence microscope (Olympus BX60, 400× objective) after staining with 4′,6-diamidino-2-phenylindole (DAPI). Briefly, after drug treatment, cells were fixed with 70% ethanol and spun onto microscopic slides and stained with DAPI (1 mg/mL) for 5 min. Cells were regarded as apoptotic based on the presence of condensed fragmented chromatin. Enlarged cells containing multiple nuclei were considered to be typical of mitotic catastrophe.

### 3.10. Annexin V/PI Dual Staining

Annexin V-FITC binding was done with an Annexin-V Fluos Staining kit (BD Biosciences, San Jose, CA, USA) according to the manufacturer’s protocol. Briefly, after drug treatment, cells (1.5 × 10^6^) were trypsinized, twice rinsed with ice-cold PBS, pelleted and resuspended in 100 µL binding buffer containing annexin V-FITC and PI. Cells were incubated for 15 min at room temperature in the dark, then diluted with 400 µL binding buffer and analyzed by flow cytometry with FACS Accuri C6 (BD, San Jose, CA, USA). Cells with low FITC and PI fluorescence were regarded as viable. Cells that presented high FITC fluorescence but low PI fluorescence were regarded as early apoptotic. Late apoptotic or secondary necrotic cells presented high FITC and PI fluorescence. Primary necrotic cells had low FITC fluorescence and high PI fluorescence.

### 3.11. Mitochondrial Transmembrane Potential Measurement

Changes in the mitochondrial membrane potential (ΔΨm) were analyzed by flow cytometry using 5,5′,6,6′-tetrachloro-1,1′,3,3′-tetraethylbenzimidazolyl-carbocyanine iodide, JC-1 (BD Biosciences, San Jose, CA, USA) according to the manufacturer’s specifications. Briefly, after treatment 1 × 10^6^ cells were trypsinized, pelleted, resuspended in media and stained with JC-1 for 15 min at 37 °C. After staining, cells were rinsed twice with assay buffer and analyzed by flow cytometry with FACS Accuri C6 (BD, San Jose, CA, USA). The loss of ΔΨm was monitored based on a decrease in JC-1 red fluorescence concurrent with an increase in green fluorescence. 

### 3.12. Data Analysis

All presented data represent the mean of the results in at least three independent experiments ± standard deviation. Values were compared using statistical analysis performed with Graph-Pad Prism 5.0 (San Diego, CA, USA). Comparisons of differences between untreated and treated groups were obtained using one-way analysis of variance (ANOVA) followed by Dunnett’s test and * *p* < 0.05, ** *p* < 0.001 and *** *p* < 0.0001 were considered significant.

## 4. Conclusions

The final effects of antitumor agents in a patient can be modulated by many factors, including drug metabolism and drug-enzyme and drug-drug interactions due to individual expression and activity of drug-metabolizing enzymes. The aim of the presented study was to evaluate the influence of increased expression of P4503A4 and UGT1A10 isoenzymes on the biotransformation and cellular response induced by antitumor acridinone derivatives C-1305 and C-1311 in human breast MCF-7 and colon HCT116 cancer cells. 

For the first time, we show that the metabolite products of C-1305 and C-1311 glucuronidation were excreted outside the tumor cell. The concentration of glucuronides inside the cell remained at a low level and the majority of these metabolites were found in the incubation media. What is more, C-1305 and C-1311 turned out to be very strong activators of P4503A4 and UGT1A10 isoenzymes, especially in HCT116 cells. Therefore, it should be considered that multidrug therapy with these compounds would result in more extensive metabolism of other drugs, which would strongly change their final effect. It is a crucial conclusion, as it relates to drug metabolism isoenzymes that are particularly important for many therapeutic compounds, P4503A4 and UGT1A10.

Studies concerning the influence of P4503A4 and UGT1A10 isoenzymes on the cellular response induced by C-1305 and C-1311 revealed that the increased expression of P4503A4 resulted in DNA degradation and induction of necrosis in a greater population of MCF-7 and HCT116 cells. The most noticeable effect was observed in C-1305-treated HCT116 cells, in which additional strong induction of apoptosis was detected but only in the first days of drug treatment. The effect of UGT1A10 on the cellular response induced by the studied compounds showed more spectacular results. Namely, our studies provide strong evidence that C-1305 after glucuronidation demonstrates higher pro-apoptotic and pro-necrotic properties. These effects were clearly observed in cells of higher UGT1A10 activity, where the glucuronidation process proceeded faster. It is worth emphasizing that the cases in which glucuronide derivative is more active than native drug are very rare and this is a next outstanding feature of C-1305. In turn, the mechanism of action of C-1311 did not change after glucuronidation and the drug maintained its anti-proliferative properties and capacity to cause cell death.

Summing up, the obtained results will allow us to predict the role of the enhanced expression of P4503A4 and UGT1A10 isoenzymes in patient responses to antitumor therapy with the studied acridinone antitumor agents. We also show the unique properties of C-1305 drug, which was glucuronidated particularly via UGT1A10 and this glucuronic form was more active than native drug. 

## Figures and Tables

**Table 1:** Activity of P4503A4 isoenzyme and UGT enzymes performing *O*-glucuronidation in selected cell lines, native and transfected (3A4/1A10) calculated as % conversion of standard substrate for P4503A4 (testosterone) and *O*-glucuronidation process (7-OH-TFC). Cells were incubated with enzyme substrate in the same conditions for the same time: 24 h for testosterone and 3 h for 7-OH-TFC.

Activity of Enzyme (% Conversion of Standard Substrate)	Cell Line					
	MCF-7	MCF-7 3A4/1A10	HCT116	HCT116 3A4/1A10	HT29	HepG2
P4503A4	0.08 ± 0.04	0.28 ± 0.04	0.10 ± 0.03	0.86 ± 0.35	*no data*	1.38 ± 0.50
UGT (*O*-glucuronidation)	0.09 ± 0.04	1.94 ± 0.62	0.01 ± 0.01	3.42 ± 0.64	59.13 ± 8.61	2.28 ± 0.53

**Table 2:** Cytotoxicity of C-1305 and C-1311 against MCF-7 and HCT116 stably transfected with empty vector (EV) cells, P4503A4 or UGT1A10 isoenzymes.

Drug Dose (µM)	C-1305			C-1311		
	MCF-7 Cells			MCF-7 Cells		
	EV	CYP3A4	UGT1A10	EV	CYP3A4	UGT1A10
IC_50_	1.87 ± 0.05	1.55 ± 0.23 *	1.57 ± 0.08 *	0.36 ± 0.08	0.23 ± 0.01 *	0.33 ± 0.04
IC_80_	9.19 ± 1.45	6.28 ± 0.73 *	6.37 ± 0.73 *	1.04 ± 0.12	0.70 ± 0.02 *	1.00 ± 0.06
	**HCT116 Cells**			**HCT116 Cells**		
	**EV**	**CYP3A4**	**UGT1A10**	**EV**	**CYP3A4**	**UGT1A10**
IC_50_	1.09 ± 0.32	1.36 ± 0.37	1.34 ± 0.15	0.96 ± 0.13	0.77 ± 0.11	1.38 ± 0.10 **
IC_80_	5.75 ± 0.89	6.53 ± 2.37	6.23 ± 0.71	5.37 ± 1.08	4.93 ± 0.82	9.31 ± 2.08 **
IC_90_	10.39 ± 1.67	26.35 ± 3.63 ***	10.77 ± 1.43	11.19 ± 1.86	9.79 ± 1.69	19.37 ± 5.86 *

Significant differences between control empty vector, EV cells and UGT1A10- or CYP3A4-transfected are indicated as follows: * *p* ≤ 0.05; ** *p* ≤ 0.001, *** *p* ≤ 0.0001.

## Abbreviations

7-OH-TFC	7-Hydroxy-4-trifluoromethylcoumarin (*O*-glucuronidation) standard substrate
ΔΨm	Mitochondrial membrane potential
DAPI	4′,6-Diaminidino-2-phenylindole (fluorescent-DNA stain)
ESI-MS	Electrospray ionization mass spectroscopy
EV	Empty vector
FITC	Fluorescein isothiocyanate (fluorescent dye)
HPLC	High-performance liquid chromatography
IC	Inhibitory concentration
RT-PCR	Reverse transcription-polymerase chain reaction
PI	Propidium iodide (fluorescent dye staining only dead cells)
UGT	UDP-glucuronosyltranspherase
